# Diagnosis of Bilateral Quadriceps Tendon Rupture Using Point-of-Care Ultrasound

**DOI:** 10.5811/cpcem.40087

**Published:** 2026-01-05

**Authors:** Edward Guo, Akaysha Duran, Alexander Kuc, Alfred B. Cheng

**Affiliations:** * Jefferson Health Northeast, Department of Emergency Medicine, Philadelphia, Pennsylvania; † University of Pittsburgh Medical Center (UPMC) Memorial, Department of Emergency Medicine, York, Pennsylvania; ‡ Cooper University Health Care, Department of Emergency Medicine, Cooper Medical School of Rowan University, Camden, New Jersey

**Keywords:** bilateral quadriceps tendon rupture, point-of-care ultrasound

## Abstract

**Case Presentation:**

A healthy 32-year-old man presented to the emergency department with bilateral knee pain after landing from a jump. He was unable to extend his knees and had pain to palpation superior to the patella. Bilateral quadriceps tendon rupture was confirmed using point-of-care ultrasound, and the patient underwent operative repair the next day.

**Discussion:**

Bilateral quadriceps tendon rupture is exceedingly rare, which often leads to misdiagnosis. Magnetic resonance imaging is the gold standard diagnostic imaging study but has multiple disadvantages, especially in emergency settings. Point-of-care ultrasound is an excellent tool to screen for this injury and prevent morbidity from delay in surgical repair.

## CASE PRESENTATION

A 32-year-old man with no past medical history presented to the emergency department (ED) for bilateral knee pain. He had been jumping on a trampoline when he landed in a squat position, felt pain in both knees, and was then unable to bear weight. He had an athletic build and body mass index of 32.1 (reference range 18.5–24.9) kilograms per square meter. Physical examination was significant for bogginess and tenderness to palpation to the distal femur bilaterally with inability to extend either knee. The patient denied medication or drug use and had no previous orthopedic surgeries.

**Image 1:**
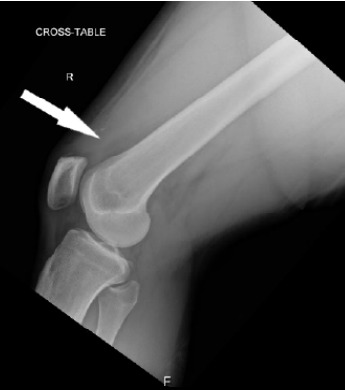
Plain radiograph of the right knee demonstrating trace suprapatellar joint fluid (arrow).

**Image 2:**
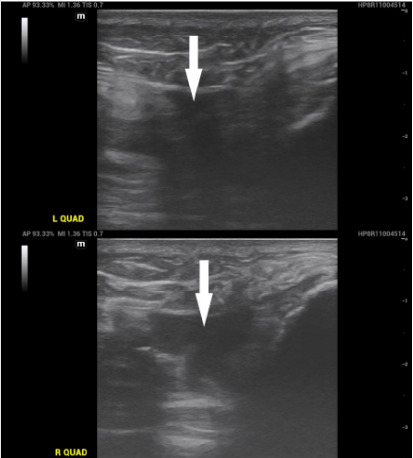
Point-of-care ultrasound of the left and right suprapatellar regions in sagittal plane demonstrating disruption in the bilateral quadriceps tendon fibers with adjacent anechoic fluid collections likely representing hematomas (arrows). *L QUAD*, left quadriceps; *R QUAD*, right quadriceps.

Plain radiographs of the bilateral knees demonstrated trace amounts of bilateral suprapatellar joint effusions with no fracture or dislocation (Image 1). Point-of-care ultrasound (POCUS) of the suprapatellar regions revealed discontinuity of the bilateral quadriceps tendons with adjacent hematomas (Image 2). Dynamic POCUS of the quadriceps tendons while the patient attempted to extend at the knee further supported a diagnosis of bilateral quadriceps tendon rupture (Video). No further diagnostic imaging studies were obtained. The patient underwent operative repair the following day and was confirmed to have complete rupture of the bilateral quadriceps tendons.

## DISCUSSION

Bilateral quadriceps tendon rupture is rare with just over 100 reported cases in the literature. It is commonly misdiagnosed at initial presentation due to its rarity and the inability to compare the affected limb to the unaffected limb.^1^ Risk factors include chronic renal disease, diabetes mellitus, obesity, and steroid use.^2^ Complete quadriceps tendon rupture is typically caused by forceful contraction of the quadriceps muscles with the knee in a flexed position while regaining balance such as in the case of our patient. Orthopedic consultation is indicated as patients suffer ambulatory dysfunction from injury to the extensor mechanism of both lower extremities. Delay in surgical repair for complete rupture is correlated with quadriceps retraction, muscle atrophy, and decreased functional outcomes.^3^

*CPC-EM Capsule*

What do we already know about this clinical entity?

*Quadriceps tendon rupture is uncommon; bilateral cases are rare and frequently missed, delaying surgical repair and worsening patient outcomes.*

What is the major impact of the image(s)?

*Video of left and right suprapatellar regions with the patient attempting to extend his knees demonstrates bilateral quadriceps tendon rupture.*

How might this improve emergency medicine practice?

*Dynamic point-of-care ultrasound may improve recognition of tendon rupture and expedite a rapid, accurate diagnosis.*

Plain radiographs may show indirect signs of quadriceps tendon rupture but are rarely diagnostic. Magnetic resonance imaging (MRI) of the knee is the gold standard imaging study but has several limitations including cost, time, and availability. Physical examination paired with radiology-performed ultrasonography has been used to diagnose quadriceps tendon rupture with sensitivities reported as high as 100%.^4^ Thus, ED POCUS is an excellent screening modality to assess for bilateral rupture and prevent delay in diagnosis, treatment, and potential morbidity. It was particularly valuable in our case due to the inability to compare findings to an unaffected limb. Furthermore, to our knowledge, no prior peer-reviewed case report has included annotated, dynamic POCUS images visualizing quadriceps tendon rupture.

Our images provide novel educational value, demonstrating a practical, real-time imaging technique to improve recognition of this rare injury. Advantages of POCUS include universal availability and rapid utility, making it an ideal screening tool for quadriceps tendon rupture in the ED that may also be diagnostic. Its major limitation is operator dependence. In cases with any doubt of the diagnosis, an MRI should be obtained given its superior specificity.^4^,^5^

## Figures and Tables

**Video:** Dynamic point-of-care ultrasound of the left and right suprapatellar regions in sagittal plane with the patient attempting to extend his knees, demonstrating bilateral quadriceps tendon rupture (arrows). *L QUAD*, left quadriceps; *R QUAD*, right quadriceps.
